# Adaptive comparison matrix: An efficient method for psychological scaling of large stimulus sets

**DOI:** 10.1371/journal.pone.0233568

**Published:** 2020-05-29

**Authors:** Isamu Motoyoshi

**Affiliations:** Department of Life Sciences, The University of Tokyo, Tokyo, Japan; University of Wuerzburg, GERMANY

## Abstract

Studies on natural and social vision often need to quantify subjective intensity along a particular dimension for a large number of stimuli whose perceptual ordering is unknown. Here, we introduce an easy experimental protocol of comparative judgments that can rank and scale subjective stimulus intensity using a comparatively small number of trials. On each trial in our protocol, the observer initially views M stimuli sampled from a space of N stimuli and selects the stimulus that elicits maximum subjective response along a given dimension (e.g., the most attractive). The selected stimulus is subsequently discarded, the observer then performs a judgment on the remaining stimuli, and the process is iterated until the last stimulus remains and a new trial begins. The method relies on sorting perceived stimulus order in the N x N comparison matrix via logistic regression and sampling the next set of M stimuli such that responses will be collected only for stimulus pairs whose expected response ratio is most informative. Numerical simulations demonstrate that this method can estimate psychological scale with a small number of responses. Psychophysical experiments confirm that the method can quickly estimate the contrast response function for gratings and the perceived glossiness of naturalistic objects. This protocol would be useful for characterizing human judgments along various dimensions, especially those with no physical image correlates such as emotional and social attributes.

## Introduction

A large number of natural images have been employed in various fields of research that study perceived properties such as object recognition [1], material perception [2], and preference for faces [3]. In such studies, observers judge the distal attributes of objects such as roughness and reflectance or perform judgments along extremely subjective dimensions such as attractiveness or social stereotype [e.g., trustworthiness: 4]. While methods such as magnitude estimation–or rating–have been used on large stimulus sets to obtain quantitative measures of subjective intensity [e.g., 5], such approaches run into practical problems. In a typical rating experiment, observers are required to continuously map a perceptual impression onto a fixed numerical scale, but the mapping criterion can easily fluctuate over trials since observers cannot anticipate the distribution of ratings *a priori* before a representative stimulus sample has been shown. In some instances, a reference stimulus 'modulus' can be used to avoid criterion fluctuations. Repeated measurements are needed for each stimulus in order to obtain reliable data.

The paired comparison is a classic method for constructing psychological scales using discrimination data derived from stimuli whose psychological order is unknown [e.g., 6]. The procedure is based on comparative judgements between stimuli that need much less effort than rating, and has been used in various fields of research, from perceptual psychology to marketing, as a basic tool for analyzing perceptual appearance and preference [7,8,9,10]. A number of studies have investigated the theoretical bases of the method in terms of signal detection and psychometrics, and proposed extended methods [e.g., 7,11,12,13,14,15].

Practically, a downside of the method is that it requires a large set of comparison judgments on several stimulus pairs. For instance, a stimulus set of N = 100 exemplars yields a space of N(N-1)/2 = 4950 possible paired-stimulus comparisons. Given that the response rate for each paired comparison must be computed over several repetitions, the total number of trials rapidly becomes too large to assign to any one individual observer. This inefficiency problem has long been recognized, and algorithms have been proposed to solve it. For example, there are some statistical methods for constructing an index from an incomplete comparison matrix [16, see also 15]. There is also a procedure proposed in which a comparison matrix is sorted on every trial, and data are gathered selectively for pairs of stimuli which are close to each other along the index; i.e., pairs near the diagonal of the matrix [17]. In the field of marketing research, studies have introduced an extended comparison method in which observers select the stimulus that elicits a maximum (or minimum) response along a given psychological dimension among multiple stimuli [e.g., 18,19,9,20]. Yet, this max-choice paradigm had largely been ignored in research of perception and cognition.

Combining the abovementioned tactics and procedures, the present study introduces an experimental protocol that meets several conflicting requirements, namely collecting response data to estimate psychological order and scale in a large stimulus set along a particular dimension while minimizing the number of trials and engaging the observer with a straightforward and comfortable task. This paper describes the basic and easily-implemented protocol of the method, the simulation results regarding the efficiency of the method, and some applications to psychophysical measurements with human observers. We also discuss theoretical and practical problems and limitations of the method.

### Method overview

The present method can be broken down into three distinct processes that can be described algorithmically as follows: (1) collect responses in a comparison task for a subset of M stimuli chosen from a set of N stimuli, (2) update and sort the comparison matrix, and (3) select a new set of stimuli for the next trial based on the sorted comparison matrix. Each of the three processes is further explained below.

#### 1—Collecting response data

**Fig 1:**
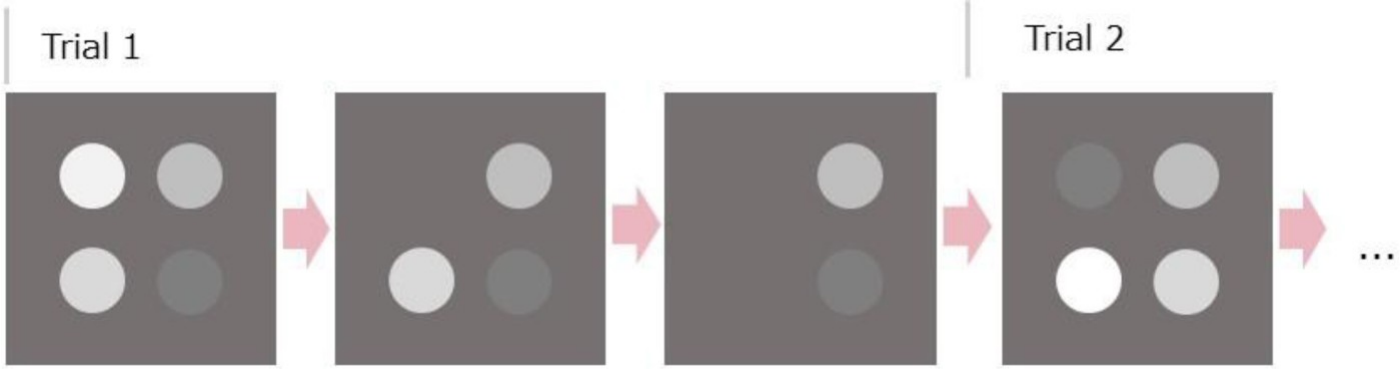
A single-trial process of iterative stimulus selection and elimination for set size M = 4. Observers select the brightest stimulus, the chosen stimulus then disappears, and the observer continues on selecting the brightest stimulus from the remaining set.

M stimuli (M> = 2) are initially presented on each trial. Stimuli are selected at random from a set of N stimuli and then further selected via the sampling rule for the second trial (described below). Instructions are given by the experimenter, and out of the M available stimuli, observers select the stimulus that elicits a maximum psychological value along a particular dimension (Fig 1). This first response indicates that the selected stimulus has a larger psychological value than all the other M-1 stimuli. For instance, if there are M = 4 visual stimuli (A, B, C, and D) on the screen, and an observer chooses A as the brightest stimulus, then A is perceived as brighter than B, C, and D by logical necessity. The key insight here is that a single response by the observer effectively yields multiple paired (M-1) comparisons– 3 paired comparisons (i.e., A-B, A-C, A-D) in our example with M = 4.

Once a response is made, the selected stimulus is taken away, or marked, and the observer then selects a new stimulus from the remaining choices. Through this process of iterative elimination, the observer continues on selecting a new stimulus until a response is obtained either for the last two remaining stimuli or until the number of responses for that trial reaches a criterion number determined by rules described below. After the last trial response is obtained, the experiment moves on to the next trial and a new set of M stimuli is selected (see rules below).

#### 2—Updating and sorting the comparison matrix

**Fig 2:**
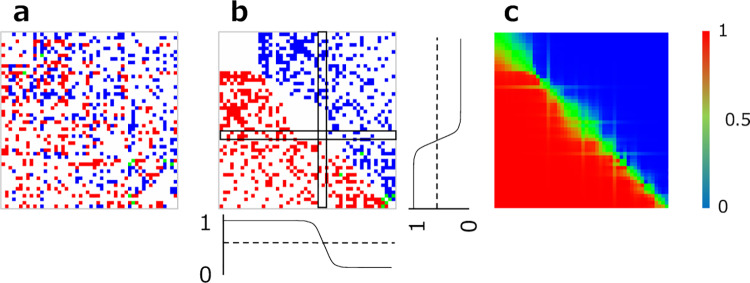
(a) An example of a comparison matrix obtained for 50 stimuli in total (N = 50). An observer has chosen stimuli 53 times up to this point. The color of each dot represents response rate (blue for low rate, red for high rate, green for a response rate of 0.5, white for no data). (b) Sorted comparison matrix. (c) A matrix of response rate estimated from psychometric functions.

A comparison matrix (Fig 2A) tabulates the "win" rate of each stimulus vs. every other stimulus. Here, we define win rate as the response rate at which a particular stimulus is reported as larger than another stimulus along a given psychological dimension (e.g., brightness). After each observer response, data are incorporated into the existing response histogram where a "1" and a "0" are added to the winning and losing stimuli in the pair. Pairwise win rates ranging from 0 to 1 are then computed from the updated response histogram.

Next, the order of the stimuli in the comparison matrix is sorted in a two-step procedure. In the first step, stimuli are sorted in a way that a stimulus which has a larger sum (P) of response rate is given a higher rank.

$$P_{i}=\sum_{j=1}^{N}p_{i,j},$$ Eq (1)

where *p* is the response rate for stimulus *i* over stimulus *j* (i≠j). For stimuli which have no response rate data up to this point, P is randomly set to be either 1 or 0. For stimuli which share the same response rate, rank is randomly decided within these stimuli. In the second step, for each stimulus (*i'*) in a sorted comparison matrix, a psychometric function (logistic function in the present study) is fitted to the response rate of all the other stimuli (*j'*) by means of a maximum likelihood method [21]. As the matrix is mirror symmetric, the two fitted logistic function are obtained along the both *i*' and *j*' axes in the sorted comparison matrix. The sigmoid curves in Fig 2B illustrate the fitted functions. The estimated response rates (q_i',j'_) are defined as the average of the two.

$$q_{i\prime ,j\prime }=\left[\frac{1}{1+exp(-a∙(i\prime -x))}+\frac{1}{1+exp(a∙(j\prime -x))}\right]/2,$$ Eq (2)

where *a* is the slope and x is the centroid of the function. In this fitting procedure, the response rate for the 0 th stimulus vs. all the others are set to be 1, and the response rate for the N+1 th stimulus vs. all the others are set to be 0. Then, stimuli are sorted in a way such that a stimulus with a larger total sum of estimated response rate (q_i',j'_) is given a higher rank in the same way as in Eq (1).

This two-step procedure is implemented repeatedly so that regression residuals are minimized. Fig 2B shows the comparison matrix sorted in the way described here. Our simulations showed that this method gave better results compared to the other computations we tested; e.g., single logistic fitting along *i'* axis only, no repetition of fitting and sorting, and so on.

3—Sampling a *n*ew *s*timulus *s*et for the *n*ext *t*rial. Lastly, a new set of M stimuli for the next trial is selected based on data from the sorted comparison matrix. Since it is redundant to present a set of stimuli which has been presented many times or which shows a clear tendency of winning against the other(s), a new set of stimuli should be selected in a way that efficiently eliminates this redundancy. This can be achieved by various approaches, but for the sake of clarity and for the purpose of demonstrating a principle and on the basis of pilot observations, the present study employed a simple index to prioritize stimuli that have less response data and whose expected win rates are closer to 0.5.

In the present study, the search for the optimal next-trial stimulus set begins with an initial set of M stimuli randomly selected out of a set of N possible stimuli. Then, a stress factor (S) is calculated for all possible stimulus pairs in M such that

$$S_{i,j}=|2p_{i,j}-1|+n_{j}/N,$$ Eq (3)

where *p*_*i*,*j*_ is the win rate (0 to 1) of stimulus *i* over stimulus *j* which we estimate from the corresponding psychometric function, and *n*_*j*_ is the total number of the responses obtained for stimulus *j*. In order to find an optimal set of M stimuli for the next trial, random stimulus sampling is carried out until the overall stress factor is minimized (up to 50000 times). In cases where the number of responses are few and overall stress is 0, random sampling simply terminates. The key property of this search algorithm is that the probability of selecting a stimulus pair near the comparison diagonal (Fig 2B) increases as data from ongoing trials accumulate. Alternative search algorithms should incorporate similar considerations in their design.

Another consideration is the number of responses that should be obtained from the next trial, and one can determine this in ways that balance experiment efficiency with the ease and comfort of the task for the observer. Based on several pilot observations, we used the ratio (or squared ratio) of total stress S to M to determine how many responses to collect during the next trial over the span of 1 to M-1. In accordance with this rule, an observer would select only one stimulus on each trial in the beginning of the experiment and would then select more and more stimuli (up to M-1) within each trial as trials go on. We have confirmed this by means of simulation (N = 100, M = 8; see below). If the rule above is not followed, and if the observer selects M-1 stimuli from the beginning, then the number of responses required to obtain a good estimation of psychological values is approximately 1.6 times larger than in the adaptive-rule scenario.

### Estimating psychological scale

With the above procedure, one can roughly infer the rank order a set of N stimuli along a given psychological dimension (e.g., brightness, preference, etc.). By itself, rank ordering is helpful to understand how observers evaluate perceptual and cognitive stimulus attributes (e.g., which face the observer likes the best) in many psychophysical studies. It might be ideal if one could also extract quantitative psychological values–or magnitudes–elicited by each stimulus.

It is important to note that scaling is an independent issue from the measurement protocol described above, and researchers should adopt the method most suited to their purpose. There are many methods to derive the scale from the complete or incomplete comparison matrix [6,16,22], some of which are based on different models of comparison. Especially when physical measurements of stimuli (e.g., luminance) are known, one can also derive the scale (e.g., brightness) based on JND (just-noticeable-difference) as estimated from the comparison data [23,24,25,26].

### Simulation

We performed numerical simulations to better measure the efficiency of our method to estimate psychological order and magnitude. We also seeked to determine how efficiency depends on the total number of stimuli (N), on the number of stimuli in each trial (M), on the maximum number of responses collected in each trial, and on the response characteristics of the system. All programs were written in C++ with homemade libraries.

## Methods

We began with a set of N stimuli, and each stimulus was assigned a number representing its position in the 1 to N sequence (i.e., all stimuli were assigned different numbers). For the purposes of the simulation, each stimulus was given a "psychological" value taken from a continuum ranging from 0 to 1 divided into equal linear increments (e.g., stimuli in a set of N = 3 would receive psychological values of 0.0, 0.5, and 1.0 whereas a set of N = 5 would receive psychological values of 0.0, 0.25, 0.5, 0.75, and 1.0) such that no two stimuli share the same psychological value. Critically, stimulus number and psychological value were randomly paired to avoid any relationship between physical and psychological quantities that could undermine the simulation.

We then defined a simple model observer whose response matched true psychological stimulus value with the addition of Gaussian noise whose default standard deviation was set at 1/20 (= 0.05) relative to the total range of the normalized model-response function (i.e., from 0 to 1). A set of M stimuli were presented on each trial, and the model observer reported the stimulus which, according to its evaluation of all M stimuli, had the largest psychological value.

**Fig 3:**
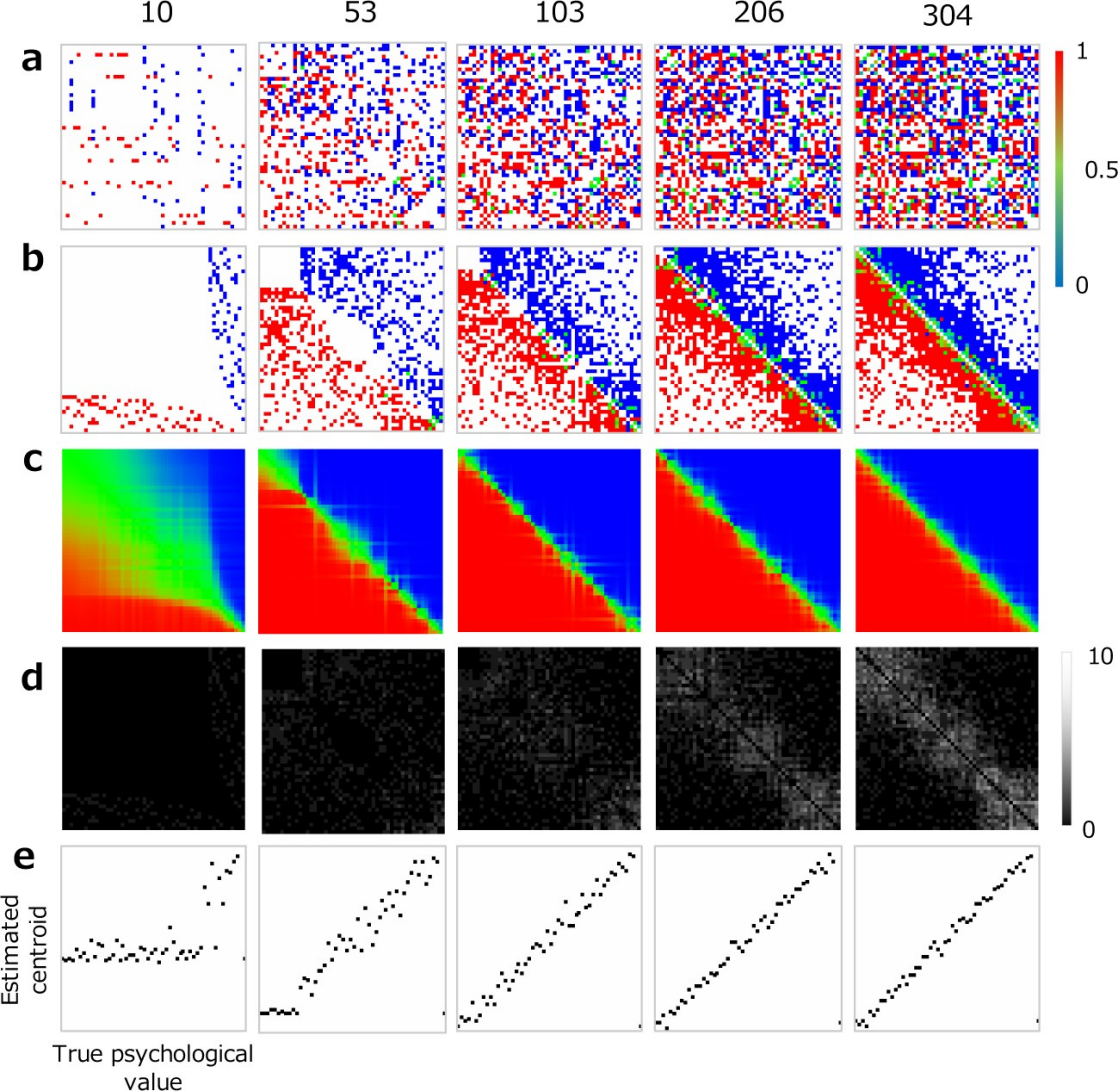
An example of simulation results with model observers. Total number of stimuli (N) is 50, and number of stimuli presented on each trial (M) is 8. Each column shows a simulation snapshot taken after a given number of model responses and shows how different metrics (rows) evolve as model responses accumulate. (a) Comparison matrix. Color of each dots represents response rate. (b) Sorted comparison matrix. (c) Matrix of response rate estimated from psychometric functions. (d) The number of responses obtained for each stimulus pair. (e) Estimated psychological value as a function of true psychological value.

We varied N (the total number of stimuli) and M (the number of stimuli presented on each trial) to examine how psychological values estimated from model responses approximate true psychological values in each experimental condition. On each trial, a set of stimuli was presented based on the protocol that we introduced above, and the psychological value over every stimulus was estimated from the observer’s response. Fig 3 illustrates the case of N = 50 and M = 8 and shows how the comparison matrix develops and evolves as model responses accumulate and, in particular, how estimated psychological value converges towards true psychological value as the number of accumulated model responses increases. It is especially evident that the correlation between psychometric functions centroids and true psychological value improves as the number of model responses increases (Fig 3E).

On each trial, we calculated the correlation coefficient between estimated psychological value and true psychological value. Here, we employed the estimated psychological value as determined by the JND-based method. The experiment was terminated when the correlation coefficient reached 0.95 or higher; we used correlation coefficient instead of RMS error as it makes it easier to see if the result is successful. For each combination of conditions of N and M, we repeated the simulation at least 20 times and calculated the average correlation coefficient and average number of model responses required for to reach a correlation coefficient of 0.95 or higher.

In this simulation and in human experiments described later, we used a JND-based method to estimate the scale. For the purpose of this method, the psychological magnitude of a stimulus occupying the last rank, (0)^th^ stimulus, in a sorted comparison matrix is set to 0. Then, a threshold between the i^th^ stimulus and the (i+1)^th^ stimulus is calculated from the slope of the fitted psychometric function, and the sum of the magnitude of the i^th^ stimulus and the threshold is defined as the magnitude of the (i+1)^th^ stimulus. Here, stimulus order (normalized to 0–1) in the sorted comparison matrix is substituted for stimulus magnitude, and threshold is defined as half of the difference between values corresponding to response rates of 25% and 75% respectively. Lastly, magnitudes are normalized to 1.

## Results

**Fig 4:**
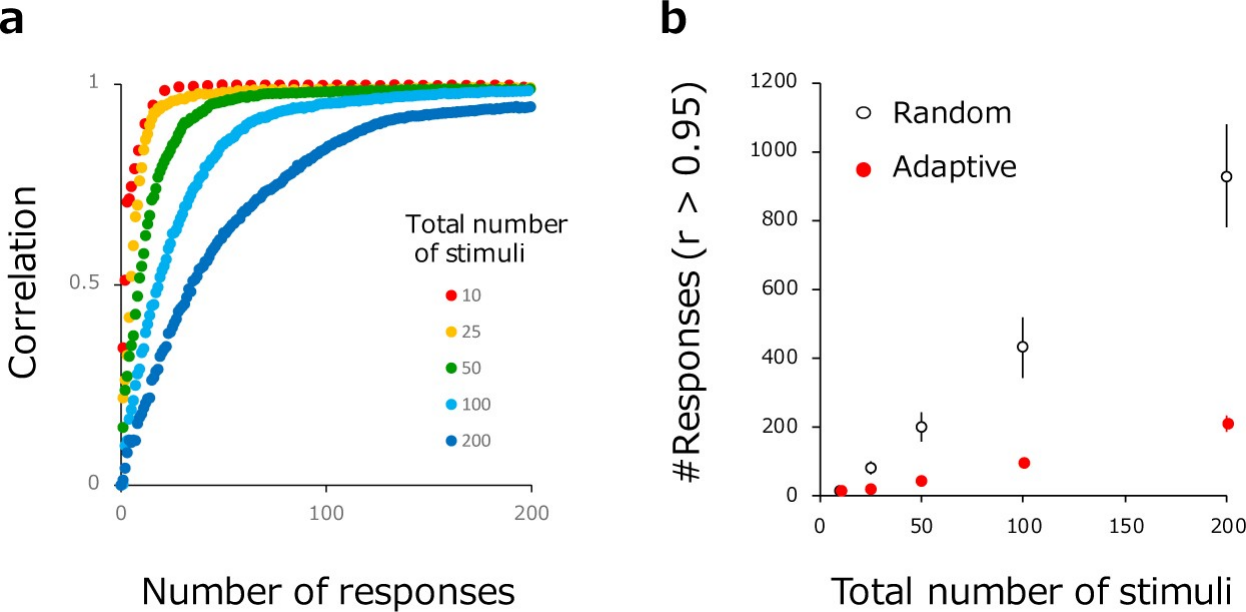
(a) Correlation coefficients between estimated and true psychological value (y-axis) as a function of number of model responses (x-axis). Colors represent different total stimulus numbers (N). (b) The number of trials required to achieve a correlation of 0.95 between estimated and true psychological value (y-axis) as a function of total stimulus number (N). Filled red circles show results for the present method. Open circles show results for the control condition (i.e., random stimulus sampling). Each dot represents an average of 20 simulation repetitions. Error bar indicates +-1SD.

Fig 4A plots the correlation between estimated and true psychological values as a function of the total number of accumulated responses. Different curves are plotted for different N's (i.e., total number of stimuli). M (the number of stimuli presented in each trial) remained fixed at 8 for all N's. As one might expect if N is small, correlation increases rapidly as responses are accumulated and reaches an extremely high value (higher than 0.95) within a handful of trials whereas correlation rises more slowly with number of responses for larger N's. Fig 4B plots the number of total responses needed for correlation to reach 0.95 for each N. Even when N exceeds 100, the present method (filled red circles) requires only 100 to 200 responses for a good estimation of psychological value. Open circles show results obtained for the control condition in which the stimulus set in the next trial was randomly sampled. Results indicate that approximately 5x as many trials would be needed without the present method's adaptive sampling. Although the required number of trials is not extremely large even in the control condition, it should be noted that this is attributable to the fact that stimulus set size (M) was 8 and the psychological values were parametrically estimated from the fitted response matrix.

**Fig 5:**
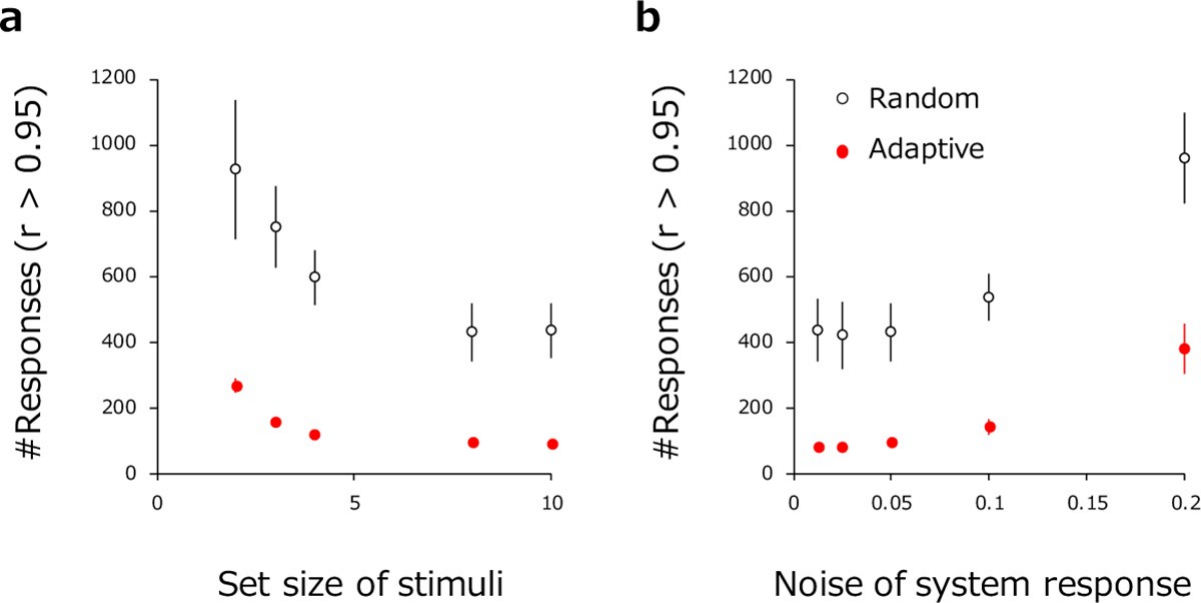
The number of trials required to achieve a correlation of 0.95 between estimated and true psychological value (a) as a function of set size of stimuli presented in each trial and (b) as a function the amount of noise in system response. Filled red circles show results for the present method. Open circles show results for the random-sampling condition. Error bar indicates +-1SD.

Fig 5A illustrates how the number of responses required for the correlation coefficient to reach 0.95 changes as the number of stimuli presented in each trial (M) increases. The plot reveals that, compared to paired comparisons (M = 2), the number of required responses in the present method (filled red circles) drops to ~1/3 for M > = 4. Again, these are far less than the required number of trials in the control condition with random sampling. Fig 5B illustrates how the number of required trials depends on the amount of noise (s.d.) in the model's response function. Here, the amount of noise is varied from very small (0.005) to very large (0.2) with respect to model's maximum normalized response (from 0 to 1). Necessarily, the number of required responses increases with the amount of noise in the model's response function. In these simulations, the total number of stimuli (N) was fixed to 100 in all conditions.

**Fig 6:**
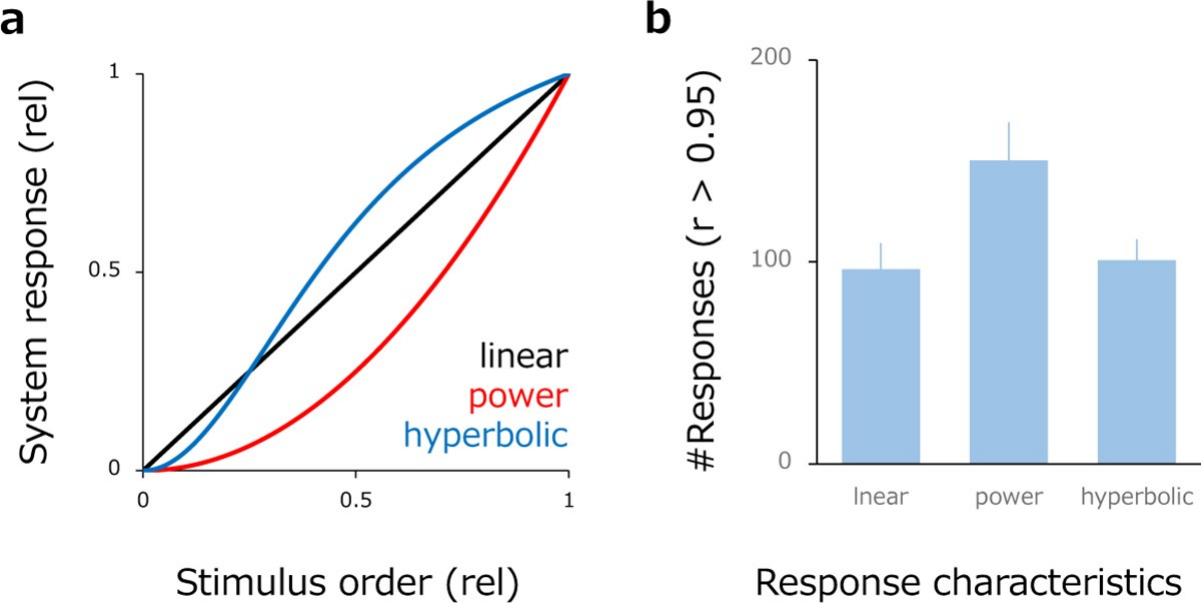
(a) Psychological value does not always increase linearly in experiments on human observers. For instance, psychological value could increase as power function (red) or as hyperbolic function (blue) of stimulus order. (b) The number of trials required for correlation between estimated and true psychological value to reach 0.95 is plotted for the three system response characteristics (linear, power, and hyperbolic) shown in (a). Total number of stimuli (N) is fixed to 100, set size of stimuli presented in each trial (M) is fixed at 8, and the amount of noise in responses is fixed at 1/20 for all three response characteristics. Error bar indicates +-1SD.

Results from the simulation above were obtained for stimuli whose psychological values increase with stimulus rank in equal linear intervals, but this is only a simplifying assumption. Indeed, for human observers, psychological differences between high-ranking stimuli is typically large while difference between low-ranking stimuli is comparatively small. Therefore, in order to precisely estimate psychological differences between stimuli, we must estimate precisely not only stimulus order but also response rates to stimuli with adjacent ranks. Fig 6 shows simulation results for cases where psychological value changes nonlinearly. Fig 6A illustrates two cases–psychological value follows either a power function (y = x^2^) or a hyperbolic function (y = x^2^ / (0.5^2^+x^2^))–and Fig 6B compares the effect of linear vs. nonlinear functions on the number of responses required to reach the 0.95 correlation criterion. Simply told, this analysis shows that nonlinear-response systems require a larger number of responses in order to properly estimate the underlying distribution of psychological values. Of course, on an *a priori* basis, one can never know exactly how psychological value increases in real-world experiments with human observers. As a general rule, then, the simulations above likely represent a lower limit on the minimum number of trials needed to determine not only the psychological stimulus order but also where each stimulus lies on a quantitative psychological scale.

In the following sections, we present two psychophysical experiments on human observers designed to examine the applicability and efficiency of our psychological scaling method. Data in these experiments were collected by an earlier version of the protocol that was a little less efficient than that described in the simulations above. The first experiment concerns the perception of luminance contrast–one of the most representative low-level image features with long and rich history of study. The second experiment concerns the perception of apparent glossiness in naturalistic objects. Unlike the first experiment, the particular interest of the second experiment is that the order of psychological values has no obvious direct physical counterpart that could be determined from the stimulus images themselves.

## Human experiment 1: Contrast perception

In a number of psychophysical studies, contrast increment thresholds (ΔC) for sinewave gratings have been measured for various pedestal contrasts C, and the relationship between ΔC and C can be used to infer the characteristics of the visual system's underlying response to image contrast [27,28]. Previous research has shown that the human contrast response function is well approximated by a hyperbolic function with few free parameters [28,29,30]. Here, we apply our psychological scaling method to estimate the contrast response function using a stimulus set with various contrast levels.

### Method

#### Stimuli

Visual stimuli consisted of 8 Gabor patches (M = 8) distributed equally along a virtual 4.9-deg radius circle. Each Gabor was composed of a vertical sine wave (spatial frequency = 1.9 c/deg) and a Gaussian window (standard deviation = 0.5 deg). The spatial phase was random. Mean luminance was 45 cd/m^2^ and matched the grey background (31.1 x 17.5 deg). We varied the luminance contrast of each Gabor over a 0.006 to 0.5 range of 20 steps (N = 20) spaced equally by 0.1 log unit increments. Stimuli were presented on a monitor (SONY PVM-A250, Japan). Luminance was gamma-corrected and controlled by a Noisy-Bit method to achieve greater than 8-bit pixel depth [31].

#### Procedure

Each trial began by presenting the eight Gabor stimuli simultaneously for 500 ms where stimulus onsets and offsets were tapered by a Gaussian window with a 167-ms standard deviation. Observers fixated a point in the center of the display and pressed a button to select the stimulus with highest perceived contrast. Following the observer's response, the selected stimulus disappeared and the remaining Gabor stimuli were presented anew with freshly randomized phases. This sequence of selecting and discarding a stimulus and subsequently displaying the remaining unselected stimuli continued until observers reached the end of the trial as determined by the rule described in the Overview section, and another set of 8 stimuli were presented to begin the next trial. Overall, each observer responded approximately 600 times or more. Five volunteers with normal or corrected-to-normal vision participated in the experiment. All experiments were approved by the Ethics Committee of the University of Tokyo. All observers provided written informed consent.

### Results

**Fig 7:**
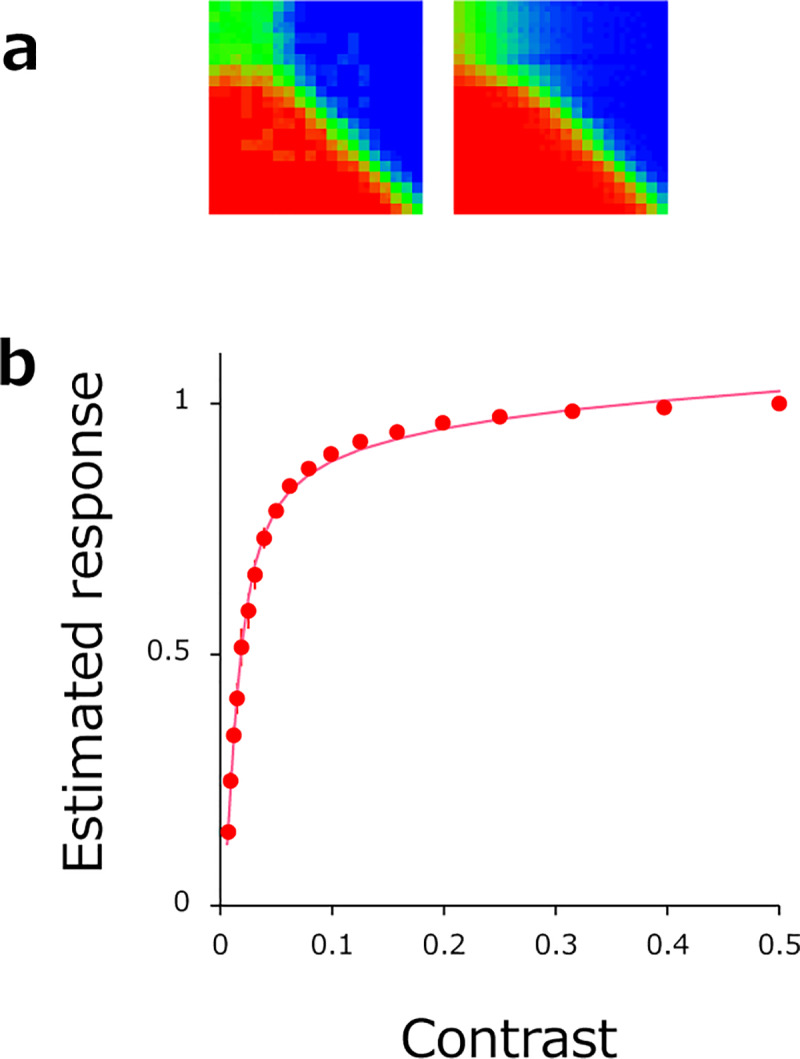
(a) A sorted comparison matrix obtained in the human contrast-discrimination task (left). and the corresponding comparison matrix estimated from fitted psychometric functions (right). (b) Estimated response magnitude as a function of stimulus contrast. Dots represents observations averaged across 5 observers. Error bars are +-1 SE (mostly invisible). The pink curve shows the best-fitting theoretical contrast response function.

Fig 7A shows a sorted (left) and fitted (right) comparison matrix obtained from a typical observer. The order of the stimuli in the matrix is equivalent to stimulus contrast values arranged on a log scale from 0.006 to 0.5 in equal steps of 0.1 log units. Fig 7B plots response magnitude as a function of physical contrast as estimated from threshold (ΔC) between adjacent contrast levels. In line with previous contrast discrimination experiments [28,29,30], the estimated contrast response function exhibits an accelerating nonlinearity at low contrasts and compressive nonlinearity at high contrasts. This nonlinear response characteristic is well approximated by the hyperbolic function R = A * C^p^ / (z^q^ + C^q^), which is displayed in Fig 7B as a smooth curve. Curve-fit parameters computed on averaged data are [A, z, p, q] = [1.08, 0.014, 1.97, 1.90] and are consistent with values reported in previous psychophysical studies (p = ~2, q = ~2). Even though the observers were under no time pressure to produce a response, the mean latency of each response was 441 (+-47) ms. Such response times are not much slower compared to those obtained in standard two-alternative choice paradigms. Our method therefore exploits another factor– time consumption – to make gains in efficiency.

## Human experiment 2: Glossiness perception

Recent psychophysical studies have investigated mechanisms underlying the perception of various material properties in natural surfaces. In such studies, various perceptual attributes such as lightness [32], glossiness [33], translucency [34,35] have been measured using matching or magnitude-estimation methods. Unlike proximal image features such as contrast or orientation, the complex attributes (e.g., lightness, glossiness, transparency, etc.) are distal in that they cannot always be defined by physical image measurements. For instance, it is usually difficult or impossible to know the correct order of physical properties such as specular reflectance of in photographs of natural real-world surfaces. Although computer-generated (CG) images afford some control over surface properties, perceived surface qualities depend in good measure on surface illumination or shape [36,37] and therefore the order of image properties cannot be determined on an *a priori* basis. Moreover, the perceptual quality originating from specular reflection is not a single-variable– or one-to-one–function but is instead characterized by two [38,39] or three [40] dimensions.

As mentioned before, the psychological scaling method we have introduced in the current paper is particularly well suited to evaluate perceived stimulus order and intensity along dimensions such as glossiness or preference that lack obvious counterparts in the physical stimulus domain. In the current experiment, we show how our psychological scaling method can be applied to the example of glossiness perception.

### Method

#### Stimuli

**Fig 8:**
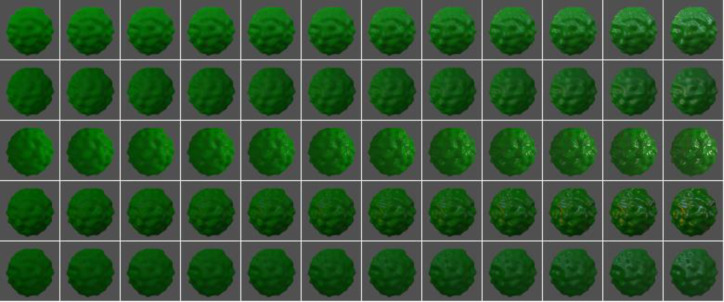
Images of object stimuli used in the experiment. Objects consisted of bumpy spheres with 12 levels of specular reflectances (columns) under 5 types of light fields (rows).

Visual stimuli comprised 60 images of objects generated by LightWave 11.6 (Fig 8). Objects consisted of bumpy spheres with 12 levels of specular reflectances illuminated under 5 types of light fields. We varied specular reflectance from 0.1 to 6.4% and applied one of 5 light fields, namely Eucalyptus, Beach, Urban11, Galileo, or Grace, taken from academic and commercial databases [41, Dosch Desigin, Germany]. The overall intensity of each lightfield was manually scaled so that resulting images appeared similar to each other and avoided excessive luminance clippings resulting from specular highlights ([x 0.8, 1.0, 1.5, 0.7, 1.0] for [Eucalyptus, Beach, Urban11, Galileo, Grace]). Each image was rendered with 1024 x 1024 pixel resolution and saved in a high-dynamic range (HDR) image format. The image background was replaced by a uniform grey. Images were anti-aliased and reduced to 256 x 256 pixels for the experiment, as this was method was effective in generating high-quality surface images. All the images were presented on a gamma corrected OEL monitor (SONY PVM-A250, Japan; 8 bit for each gun) as calibrated by a colorimeter (ColorCal, Cambridge Research Systems, U.K.). Pixels that exceeded maximum luminance were clipped.

#### Procedure

On each trial, we presented 9 objects (M = 9) on a 3 x 3 grid centered on the display. Each object image was randomly chosen from 6 possible viewing angles (objects from different viewing angles were treated as belonging to the same stimulus condition). Observers viewed stimuli freely and selected the object with the highest perceived glossiness using a button press. After each response, the chosen stimulus disappeared and observers selected the stimulus with the highest perceived glossiness among the remaining stimuli. This process was repeated until the end of the trial. Each new trial began with a new set of 9 stimuli appearing on the display. The 9 new stimuli were chosen according to the algorithm described previously. Seven volunteers with normal or corrected-to-normal vision participated in the experiment. Each observer provided anywhere between 450 and 3000 responses. All experiments were approved by the research ethics committee at the University of Tokyo and consent forms were completed.

### Results

**Fig 9:**
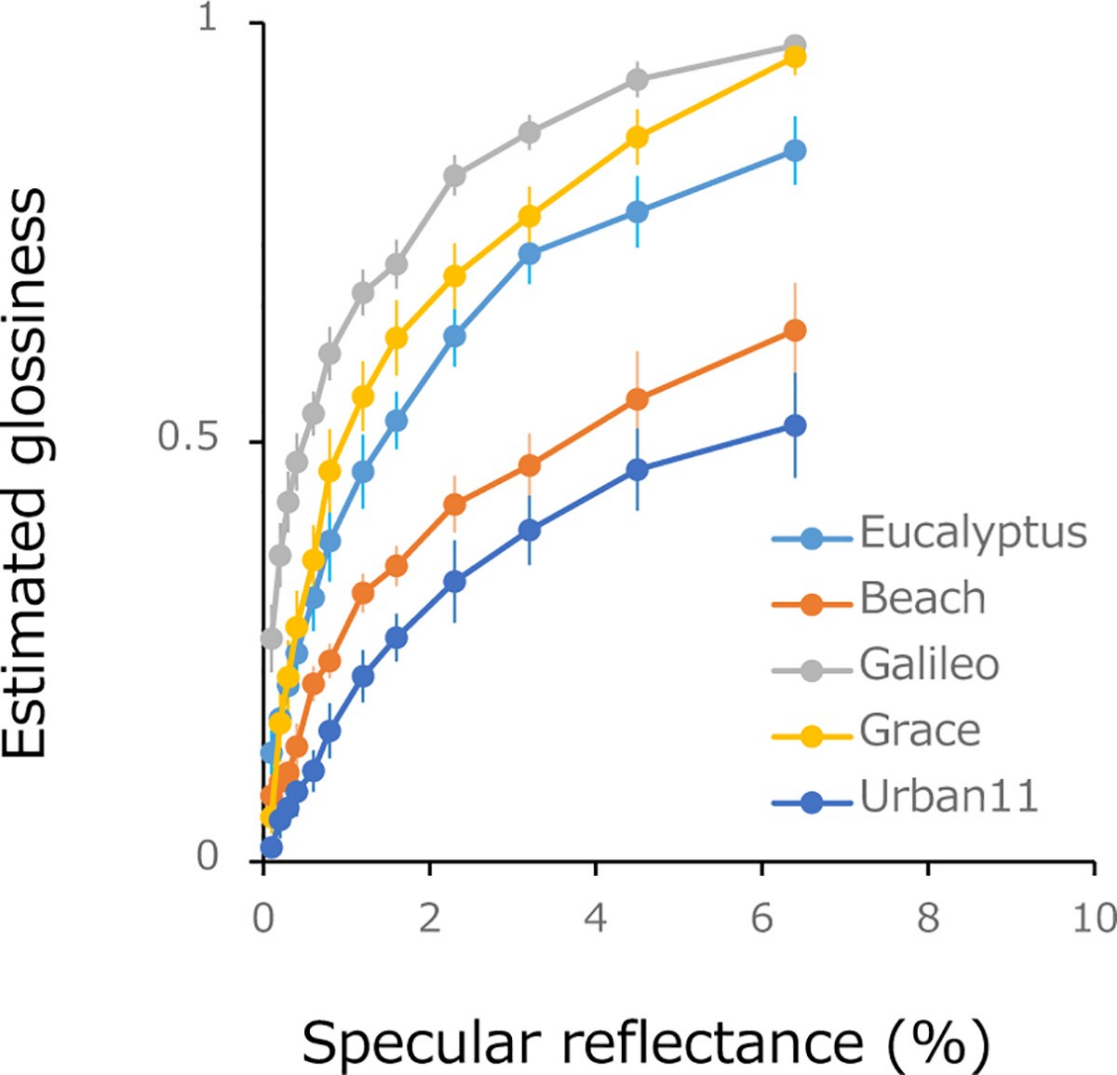
Estimated glossiness as a function of specular reflectance. Each color corresponds to objects under different light fields. Error bar indicates +-1 SE across observers.

Fig 9 plots estimated glossiness as a function of physical specular reflectance. The figure reveals that perceptual glossiness increases with specular reflectance but, in line with previous studies, also depends heavily on the type of lightfield [42,32,37] For all lightfields, estimated glossiness increases rapidly at low physical specular reflectances and saturates at high reflectances – a finding that, at least on a qualitative basis, is indicative of a non-linear glossiness response function. The differences in the estimated glossiness are qualitatively consistent with the other data obtained with psychophysical asymmetric matching [36]. The mean latency of each response was 1103 (+-494) ms. This is longer than the reaction time of ~550 ms in the two-alternative material categorization task under time pressure [43], but it is not much slower given that our observers were allowed to take as much time as they needed to discriminate the subtle difference in glossiness.

## Discussion

The current study proposes a practical experimental protocol for efficiently estimating psychological value along a certain dimension in a large stimulus set. On each trial, our method begins by presenting M stimuli (i.e., two or more) and tasks the observer with selecting the stimulus that elicits a maximal perceptual response. That stimulus is then discarded, and the observer then selects the maximum-response stimulus from the remaining M-1 set. The method thus iterates until the trial is terminated or until the last comparison involves only a pair of stimuli (M = 2). The key to our method is that each response made by the observer necessarily involves M-1 pairwise comparisons. Given that each observation carries substantially more information on perceived stimulus order and value, our method converges on psychophysical scale in significantly fewer trials than classic paired-comparison methods. Indeed, results from simulations show that the quantitative psychological scale for a space of 100 stimuli can be reasonably approximated with as little as 120 to 150 trials assuming a moderately idealized, quasi-linear, response system. Such a gain in efficiency should be especially beneficial in scenarios where a large number of samples is necessary to measure a particular perceptual scale for highly dimensional stimuli such as faces and materials. This principle is all the more true if our method is combined with other adaptive methods specifically designed to reveal the perceptual dimension itself [39,38]. It should be noted however that such high efficiency is now always expected in actual experiments in which stimuli are often unequally spaced in the psychological dimension. In practical, the efficiency could be further improved by perfecting the search algorithm used to estimate stimulus order on each trial and of to select a set of stimuli for the next trial. For example, the number of necessary trials could be further reduced by incorporating prior knowledge in the form of prior data from preceding observers or from pilot experiments.

It should be also noted that, from theoretical viewpoints, the behavioral task employed in the present method is defined as 'ranking' rather than the repetition of m-alternative forced choice. In such a task, the earlier choices could influence the later choices within a trial, and result in a little inaccurate estimation of the scale in some cases. In spite of this theoretical problem, we believe that the present method would be useful to estimate a sufficiently reliable scale in many psychophysical measurements, as demonstrated in our behavioral experiments. The experimenter may avoid this problem by using a simple max-choice task in which the observer selects only the maximum stimulus on each trial. This is statistically less efficient than the present method, but it can be practically efficient in terms of the total time and efforts in the actual experiment by introducing an adaptive control of the number of stimuli (M). In general, observers can easily and rapidly rank, or choose the max among M stimuli (e.g., M = 10) in early trials, but they would need more efforts and time to perform the task as in later trials in which M stimuli become hard to discriminate from each other (sometimes it would take 5–10 sec to select the max). If M is adaptively varied depending on task difficulty (e.g., M = 10 in easy trials and M = 2 or 3 in hard trials), observers could perform the task with a relatively constant difficulty, and need less total effort and time to complete the experiment. The rule of adaptive control would be derived from empirical data such as reaction times and the mental effort subjectively assessed by observers.

Methods based on binary comparisons between stimuli, which include our method as well as classic paired-comparison methods, can estimate the order of psychological value but faces limitations when estimating precise quantitative psychological differences between stimuli. Consider the case of four stimuli, A, B, C, and D whose luminances are 1, 10, 100, and 1000 cd/m^2^ respectively. While these stimuli can be easily discriminated–the order of psychological values is the descending D, C, B, A rank series– the discrimination task is too easy to properly infer underlying psychological stimulus values. In the above example, an observer will always produce identical responses on comparison repeats, and response rates in a comparison matrix would therefore remain pinned at 0 or 1 rather than converge on some intermediate more informative value. Mathematical differences in psychological values between adjacent stimuli that are spaced too far apart would reach ±infinity and, in the process, make it impossible to approximate the true underlying psychological scale. Therefore, in order to properly estimate psychological scale, our method must necessarily involve stimuli that are sufficiently close along the psychological dimension of interest to present a significant task challenge to observers. This would allow for natural statistical fluctuations in observer responses to map out the most informative portions of the comparison matrix with enough resolution. The other way that may overcome limitations of methods involving binary comparisons is for observers to explicitly report perceived differences between compared stimuli, as has been done in Scheffe's modified paired comparison method [7].

The objective in various fields of research often consists in searching a large stimulus set for the exemplar that elicits a maximum response along a certain psychological dimension (e.g., the most attractive face) rather than in mapping out the entire psychological scale in detail. Our method, and its modified version, can be used to efficiently find the maximum with little modification, and indeed we are currently adapting our method to not only search for the maximum exemplar in an existing stimulus set and but also generate the optimal exemplar on the fly along one or more psychological dimensions of interest.
